# BRAF-Mutated and Morphologically Spitzoid Tumor Arising Within a Congenital Melanocytic Nevus in a Pediatric Patient: A Diagnostic Challenge

**DOI:** 10.7759/cureus.104969

**Published:** 2026-03-10

**Authors:** Noria AlFadhel, Reem AlQusaimi, Fawziah AlRujaib, Munirah AlEnezi, Abdullah Alkhars, Tareq Mohammad, Fatemah Mohammad

**Affiliations:** 1 Dermatology, Mubarak AlKabeer Hospital, Kuwait City, KWT; 2 Dermatology, Abdulkareem AlSaeed Dermatology Center, Kuwait City, KWT; 3 Dermatology, AlJahra Hospital, Kuwait City, KWT; 4 Histopathology, Jaber AlAhmad Hospital, Kuwait City, KWT

**Keywords:** braf-mutated morphologically spitzoid tumor, braf v600e, congenital melanocytic nevus, immunohistochemistry staining, spitzoid melanocytic tumor

## Abstract

B-Raf proto-oncogene, serine/threonine kinase (BRAF)-mutated and morphologically spitzoid tumors (BAMS) are a recently described subset of melanocytic neoplasms characterized by spitzoid histomorphology in the presence of canonical BRAF mutations, most commonly BRAF V600E (valine-to-glutamic acid substitution at codon 600). These lesions may clinically and histologically mimic Spitz nevus, atypical Spitz tumor, or spitzoid melanoma, creating diagnostic challenges. We report the case of an 11-year-old previously healthy female with a congenital melanocytic nevus who developed a new papular lesion within the nevus. Histopathologic evaluation revealed a spitzoid melanocytic proliferation with atypical features. Immunohistochemistry supported melanocytic differentiation with a low proliferative index, and molecular testing detected a BRAF V600E mutation. An integrated clinicopathologic and molecular assessment established the diagnosis of BAMS. The patient underwent complete surgical excision with planned close follow-up. This case highlights the importance of molecular testing in spitzoid melanocytic lesions and emphasizes the need for accurate classification to guide appropriate management.

## Introduction

Spitzoid melanocytic neoplasms comprise a heterogeneous group of lesions ranging from benign Spitz nevi to atypical Spitz tumors and spitzoid melanoma. These tumors are traditionally associated with kinase fusions or Harvey rat sarcoma viral oncogene homolog (HRAS) mutations, distinguishing them from conventional melanocytic nevi and melanoma [1].

Recently, B-Raf proto-oncogene, serine/threonine kinase (BRAF)-mutated and morphologically spitzoid tumors (BAMS) have been described as a distinct subset of melanocytic neoplasms demonstrating spitzoid morphology in the presence of canonical BRAF mutations, most commonly BRAF V600E. This entity challenges the traditional molecular classification of spitzoid tumors and may clinically and histologically closely mimic other spitzoid lesions [2]. Large-scale genomic studies, including The Cancer Genome Atlas (TCGA), have demonstrated that cutaneous melanoma can be classified into molecular subtypes based on key driver mutations, most commonly BRAF, NRAS, NF1, and triple-wild-type tumors lacking mutations in BRAF, NRAS, and NF1, highlighting the central role of oncogenic signaling pathways in melanocytic tumorigenesis [3].

Given the diagnostic and therapeutic implications, accurate classification requires integration of histopathologic and molecular findings. We present a pediatric case of BAMS arising within a congenital melanocytic nevus (CMN) to highlight diagnostic considerations and management.

## Case presentation

An 11-year-old previously healthy female with a known CMN over the left elbow since birth presented with a one-year history of a newly developed skin-colored papule arising within the nevus. The background CMN had remained stable for several years without prior surgical intervention or manipulation. The new lesion was preceded by minor trauma and was noted to progressively enlarge over the course of one year. It measured approximately 1 cm in diameter and was reported to bleed easily with friction or trauma. There was no history of spontaneous ulceration. The patient’s systemic review was unremarkable, and there was no family history of melanoma or other skin malignancies.

**Figure 1:**
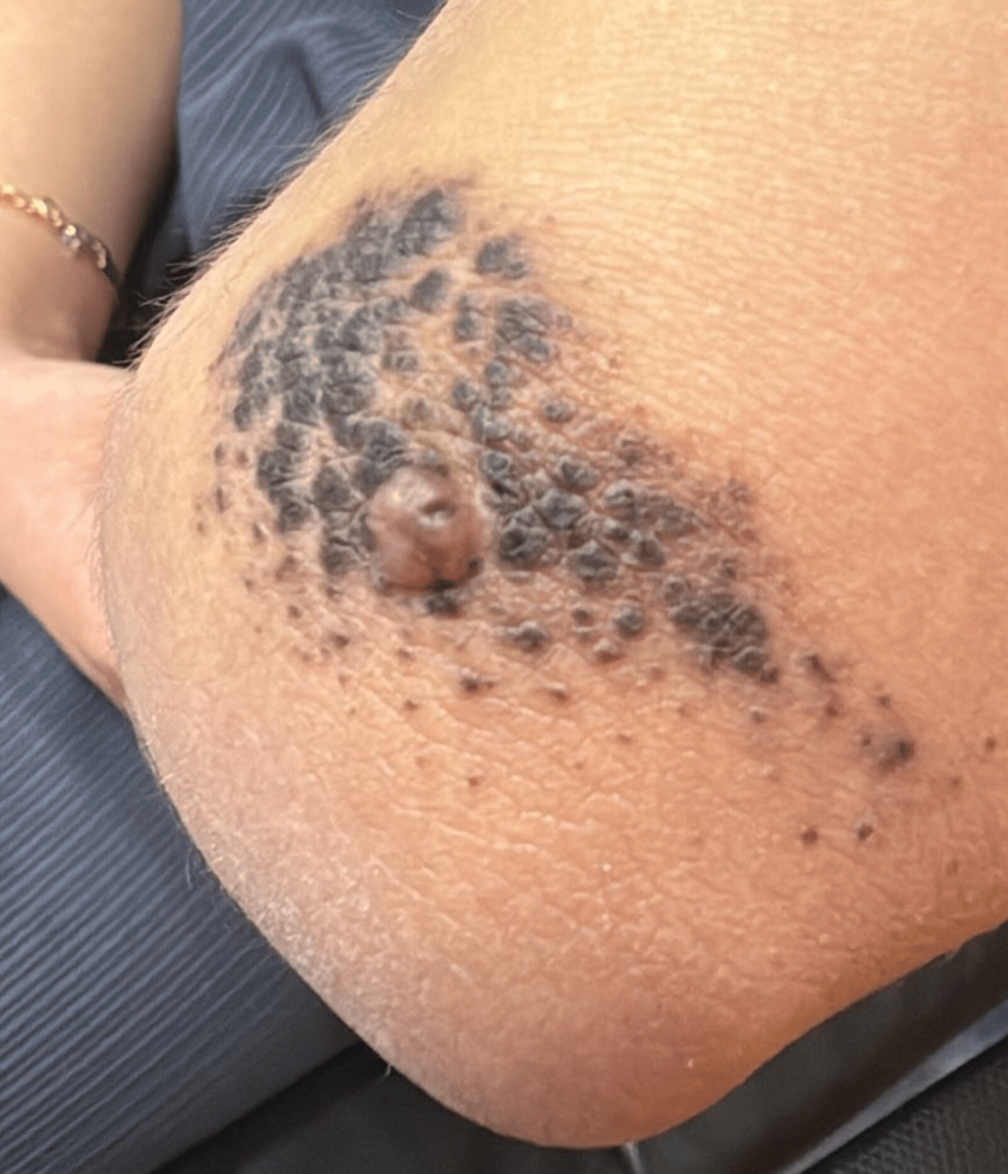
Clinical presentation at initial evaluation Clinical image of the left elbow showing a dome-shaped, skin-colored papule arising within a background CMN. CMN: congenital melanocytic nevus

On clinical examination, a dome-shaped, skin-colored papule measuring approximately 1 cm was identified on the left elbow, clearly distinct from the surrounding congenital nevus, which measured approximately 12 × 6 cm and had a papillomatous surface (Figure 1).

**Figure 2:**
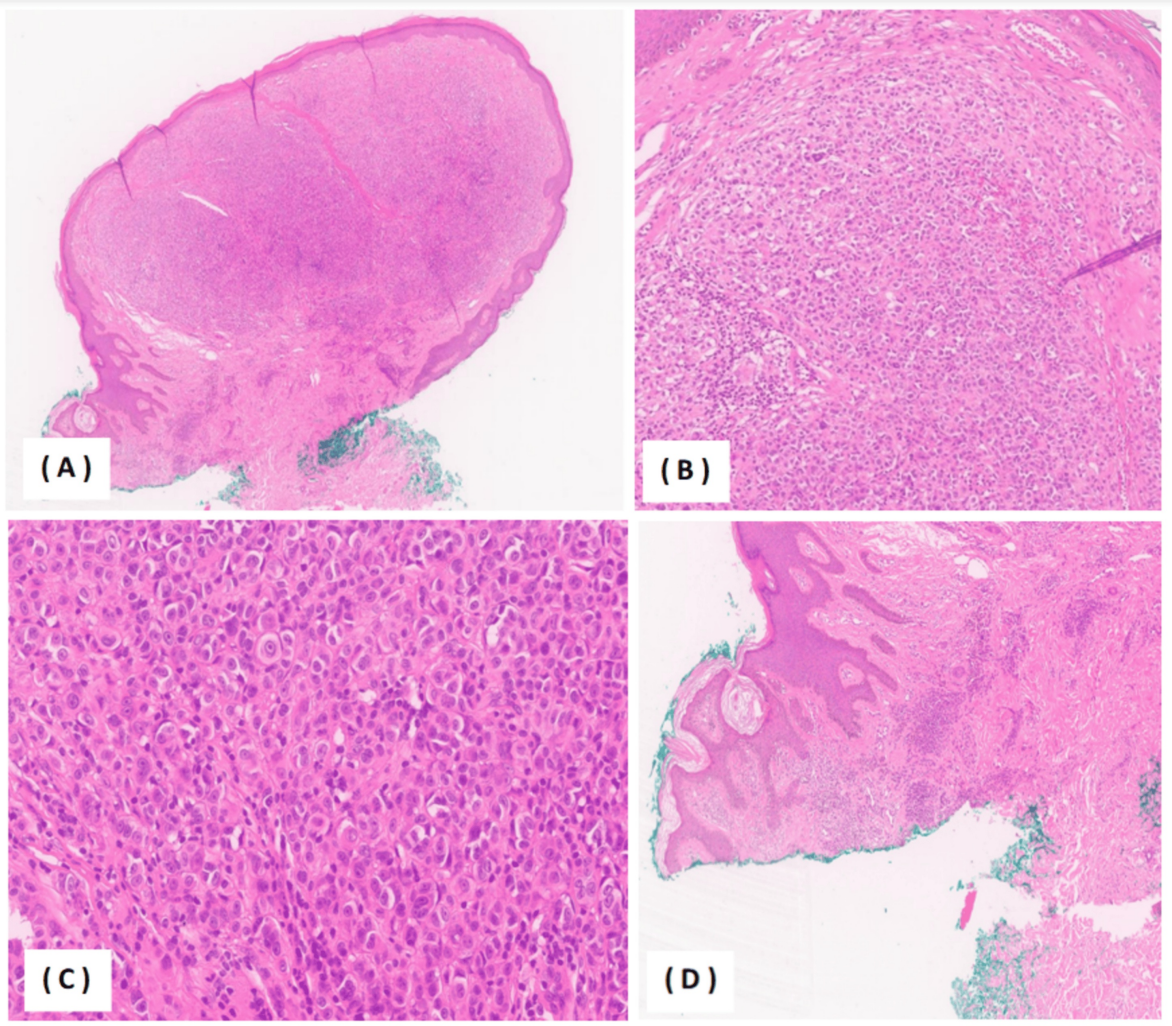
Histopathologic features of the BRAF-mutated morphologically spitzoid tumor (H&E staining) (A) Low-power H&E stain showing an asymmetrical melanocytic proliferation composed of two distinct melanocytic populations. (B) Intermediate-power H&E image demonstrating expansile, sheet-like intradermal melanocytic proliferation. (C) At higher magnification, the atypical component consisted of epithelioid melanocytes exhibiting mild-to-moderate cytologic atypia, vesicular nuclei, and prominent nucleoli. (D) First population: lentiginous junctional growth with bland intradermal nested melanocytes beneath a papillomatous epidermis. BRAF: B-Raf proto-oncogene, serine/threonine kinase, H&E: hematoxylin and eosin

**Figure 3:**
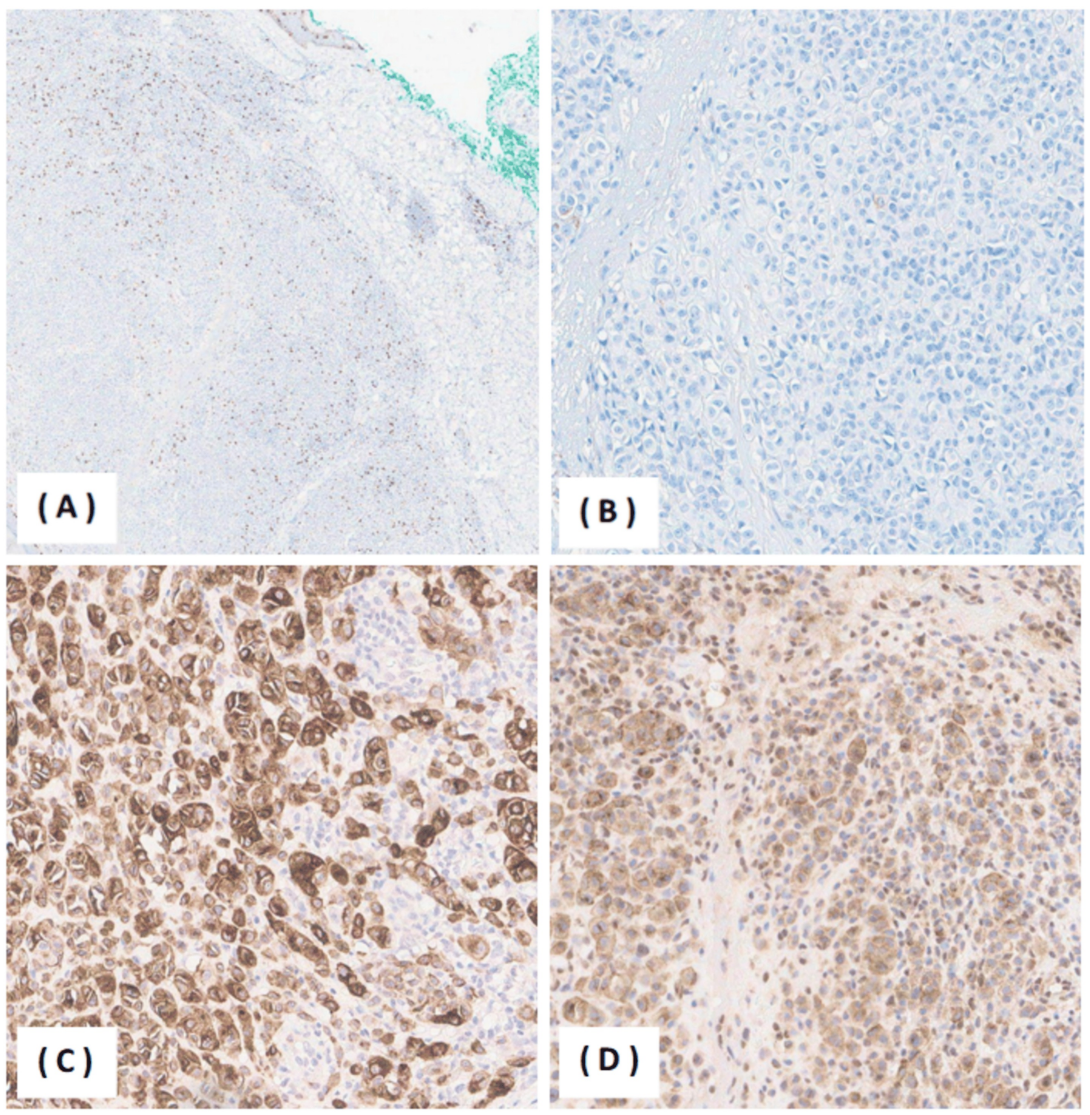
Immunohistochemical profile of the lesion (A) Ki-67 immunostain demonstrating a low proliferative index (approximately 5-7%), predominantly confined to superficial tumor cells. (B) HMB-45 immunostain negative in tumor cells. (C) S100 and Melan-A immunostains showing diffuse positivity, confirming melanocytic differentiation. (D) BAP-1 immunostain demonstrating cytoplasmic staining with equivocal nuclear loss; p16 showing patchy nuclear positivity.

Laboratory investigations were unremarkable. The patient was referred for a punch biopsy. Histopathologic examination demonstrated an asymmetrical melanocytic lesion composed of two distinct melanocytic populations. The first component involved the dermal-epidermal junction and showed predominantly lentiginous junctional growth with bland intradermal nested melanocytes beneath a papillomatous epidermis. The second component revealed an atypical intradermal melanocytic proliferation with expansile, sheet-like architecture. The Breslow thickness measured 1.9 mm, corresponding to Clark level IV. At higher magnification, the atypical component consisted of epithelioid melanocytes exhibiting mild-to-moderate cytologic atypia, vesicular nuclei, and prominent nucleoli. The mitotic rate was up to 4/mm². No definite pagetoid spread, lymphovascular invasion, or ulceration was identified. Tumor-infiltrating lymphocytes were non-brisk (Figure 2). Immunohistochemical studies showed positivity for S100 and Melan-A, while HMB-45 was negative in tumor cells. Ki-67 demonstrated a low proliferation index (approximately 5-7%). BAP-1 showed cytoplasmic staining with equivocal nuclear loss (internal control not available), and p16 exhibited patchy positivity (Figure 3). PRAME was not contributory. Molecular testing detected a BRAF V600E mutation, with no additional gene rearrangements or mutations identified.

Based on the integrated clinical, histopathologic, immunohistochemical, and molecular findings, the lesion was diagnosed as BAMS. The patient was referred to the National Bank of Kuwait Specialized Hospital for Children for multidisciplinary evaluation and was subsequently referred to the Al Babtain Center for complete surgical excision. Excision with an additional 1 cm margin was recommended, followed by close clinical follow-up.

## Discussion

BAMS are a distinct subset of melanocytic neoplasms characterized by canonical BRAF mutations, most commonly BRAFV600E, occurring in lesions that histologically mimic spitzoid morphology. While Spitz nevi and related tumors traditionally harbor kinase fusions or HRAS mutations as their primary drivers, BAMS lesions exhibit spitzoid features despite their molecular profiles aligning more closely with those of conventional melanocytic nevi and melanomas [1]. Clinically, BAMS typically presents as solitary, symmetric cutaneous papules or nodules that can occur across a broad age range, from children to adults, and may resemble benign Spitz nevi or more worrisome spitzoid melanomas [2].

At the molecular level, BAMS is driven by activating BRAF mutations, most commonly BRAF V600E, which result in constitutive activation of the MAPK (RAF-MEK-ERK) signaling pathway and promote melanocytic proliferation and survival. Although BRAF mutations are classically associated with conventional acquired nevi and cutaneous melanoma, their presence in tumors with spitzoid architecture highlights that morphologic phenotype does not strictly correlate with oncogenic driver mutations [1].

The mechanisms underlying the development of spitzoid cytomorphology in BRAF-mutated lesions remain incompletely understood. It has been proposed that additional genetic events or epigenetic modulation may influence tumor phenotype. As with other BRAF-mutated melanocytic proliferations, oncogene-induced senescence may account for indolent behavior in some cases, whereas progression likely requires secondary alterations, such as TERT promoter mutations, CDKN2A loss, or chromosomal instability [3]. These findings suggest that BAMS exists along a biological spectrum rather than representing a single uniform entity.

Specific risk factors unique to BAMS have not been clearly established. Given the high prevalence of BRAF mutations in acquired melanocytic nevi and melanoma, intermittent ultraviolet (UV) exposure is considered a potential contributing factor, particularly in younger individuals. However, direct causal evidence linking UV radiation specifically to BAMS is lacking [3].

The diagnosis of BAMS requires integrating histopathologic and molecular findings. Histologically, these lesions show spitzoid architecture composed of epithelioid and/or spindle melanocytes with abundant cytoplasm and prominent nucleoli. Architectural symmetry and maturation may be present in more indolent lesions, whereas cytologic atypia and mitotic activity can vary along the biological spectrum [4].

Immunohistochemistry confirms a melanocytic lineage (S100, SOX10, and Melan-A) but is not diagnostic on its own. Ki-67 may assist in assessing proliferative activity, and diffuse PRAME expression may raise concern for melanoma in an appropriate morphologic context. Definitive classification relies on molecular testing demonstrating a canonical BRAF mutation, most commonly BRAF V600E, in the absence of the kinase fusions typical of conventional Spitz tumors [1].

The primary differential diagnoses of BAMS include Spitz nevus, atypical Spitz tumor, and spitzoid melanoma. Spitz nevus is typically symmetric and well circumscribed, most commonly affecting children, and is usually driven by kinase fusions or HRAS mutations rather than canonical BRAF mutations. Atypical Spitz tumor exhibits worrisome features such as asymmetry, increased mitotic activity, or incomplete maturation. It represents an intermediate lesion with uncertain biological potential, in which molecular profiling is particularly helpful. Spitzoid melanoma demonstrates marked cytologic atypia, frequent or atypical mitoses, ulceration, lack of maturation, and often chromosomal instability. However, BRAF mutations may be present; diagnosis relies on overall architectural disorder, proliferative index, and genomic abnormalities rather than mutation status alone [5].

Management of BAMS is primarily surgical and depends on histopathologic risk assessment. For lesions without high-risk features, complete excision with negative margins is generally considered adequate treatment. In cases demonstrating significant atypia, increased mitotic activity, or other concerning features, wider excision may be considered, guided by principles applied to atypical Spitz tumors or melanoma [4].

Sentinel lymph node biopsy is not routinely recommended but may be considered selectively in lesions with worrisome characteristics. Given the evolving understanding of biological behavior, clinical follow-up is advised, particularly for tumors with intermediate or uncertain malignant potential [6].

## Conclusions

BAMS represent an emerging subset of melanocytic neoplasms that challenge traditional molecular and morphologic classifications of spitzoid lesions. Their significant clinical and histopathologic overlap with Spitz nevus, atypical Spitz tumor, and spitzoid melanoma underscores the importance of integrating molecular testing into diagnostic evaluation. Accurate recognition of BAMS is essential to avoid both overtreatment and undertreatment, particularly in pediatric patients. Continued reporting of well-characterized cases is necessary to further clarify the biological behavior and optimal management of this evolving entity.
